# MicroRNA Expression Profiling on Paired Primary and Lymph Node Metastatic Breast Cancer Revealed Distinct microRNA Profile Associated With LNM

**DOI:** 10.3389/fonc.2020.00756

**Published:** 2020-05-19

**Authors:** Ramesh Elango, Khalid A. Alsaleh, Radhakrishnan Vishnubalaji, Muthurangan Manikandan, Arwa M. Ali, Nashwa Abd El-Aziz, Abdulrhaman Altheyab, Ammar Al-Rikabi, Musaad Alfayez, Abdullah Aldahmash, Nehad M. Alajez

**Affiliations:** ^1^Cancer Research Center, Qatar Biomedical Research Institute (QBRI), Hamad Bin Khalifa University (HBKU), Qatar Foundation (QF), Doha, Qatar; ^2^Department of Medicine, Oncology Center, College of Medicine, King Saud University, Riyadh, Saudi Arabia; ^3^Stem Cell Unit, Department of Anatomy, College of Medicine, King Saud University, Riyadh, Saudi Arabia; ^4^Medical Oncology Department, Prince of Wales Hospital, Randwick, NSW, Australia; ^5^Department of Medical Oncology, South Egypt Cancer Institute, Assiut University, Assiut, Egypt; ^6^Division of Hematology-Oncology, Oncology Center, King Saud University Medical City, King Saud University, Riyadh, Saudi Arabia; ^7^Department of Pathology, King Saud University Medical City, Riyadh, Saudi Arabia

**Keywords:** breast cancer, lymph node metastasis, miRNA signature, hsa-miR-205-5p, hsa-miR-214-3p

## Abstract

Breast cancer (BC) is the foremost cause of cancer-related deaths in women. BC patients are oftentimes presented with lymph node metastasis (LNM), which increases their risk of recurrence. Compelling data have recently implicated microRNAs in promoting BC metastasis. Therefore, the identification of microRNA (miRNA)-based molecular signature associated with LNM could provide an opportunity for a more personalized treatment for BC patients with high risk of LNM. In current study, we performed comprehensive miRNA profiling in matched primary breast and LNM and identified 40 miRNAs, which were differentially expressed in LNM compared to primary tumors. The expression of 14 miRNAs (Up: hsa-miR-155-5p, hsa-miR-150-5p, hsa-miR-146a-5p, hsa-miR-142-5p and down: hsa-miR-200a-3p, hsa-miR-200b-3p, hsa-miR-200c-3p, hsa-miR-205-5p, hsa-miR-210-3p, hsa-miR-214-3p, hsa-miR-141-3p, hsa-miR-127-3p, hsa-miR-125a-5p, and hsa-let-7c-5p) was subsequently validated in a second cohort of 32 breast and 32 matched LNM tumor tissues. Mechanistically, forced expression of hsa-miR-205-5p, or hsa-miR-214-3p epigenetically inhibited MDA-MB-231 cell proliferation, colony formation, and cell migration. Global gene expression profiling on MDA-MB-231 cells overexpressing hsa-miR-205-5p, or hsa-miR-214-3p in combination with *in silico* target prediction and ingenuity pathway analyses identified multiple *bona fide* targets for hsa-miR-205-5p, hsa-miR-214-3p affecting cellular proliferation and migration. Interestingly, interrogation of the expression levels of hsa-miR-205 and hsa-miR-214 in the METABRIC breast cancer dataset revealed significantly poor overall survival in patients with downregulated expression of miR-205 [HR = 0.75 (0.61–0.91)], *p* = 0.003 and hsa-miR-214 [HR = 0.74 (0.59–0.93) *p* = 0.008]. Our data unraveled the miRNA-transcriptional landscape associated with LNM and provide novel insight on the role of several miRNAs in promoting BC LNM, and suggest their potential utilization in the clinical management of BC patients.

## Introduction

Despite the advances in cancer management, cancer remains the second foremost cause of death globally. Breast cancer is the most prevalent malignant disease among women, with projected 2.1 million newly diagnosed breast cancer cases and close to 626,679 associated deaths in 2018 worldwide (1). Breast cancer incidence is likely to become a significant healthcare burden with increasing population and aging worldwide, as well as in the gulf region. The age-standardized incidence rates for BC are increasing in several countries, including the Arab region where the observed BC incidence were 9·5–50 cases for every 100,000 women per year. While the reported incidence for BC in this Arab region is less than incidence observed in other geographic regions, the incidence of BC in this region is increasing (1, 2). BC patients in the Arab world are usually presented with larger and more aggressive tumors and nearly a decade younger than BC patients from other regions (3). A comparative study of BC patients from Lebanon, Tunisia, and Morocco (south Mediterranean) and France (North Mediterranean) revealed tumors with a more aggressive clinical and pathological features in the south Mediterranean group, which were mostly luminal B, compared to the north BC which were predominantly luminal A molecular subtype (4).

Better understanding of breast cancer lymph node metastasis (LNM) and the identification of novel molecular signature are essential for better disease stratification and management choices. Tumor metastasis is a complex process which is initiated by the spreading, seeding, and subsequent engraftment of malignant breast cancer cells in remote sites (5–7) is the leading cause of breast cancer-related death (8). It is well-known that BC patients with LNM are more likely to exhibit greater invasive clinical presentation at the time of diagnosis than patients without lymph node metastases. BC LNM could be influenced by several factors, including transcriptional factors, chemokines and cellular receptors, and subsequent changes in cytoskeleton organization, adhesion, and directional migration (9). Studies have shown that increased levels of chemokine receptors in lymph nodes, lung, liver, breast, and bone marrow-organs are related to tumor metastasis (10).

MicroRNAs (miRNAs) are small non-coding RNA molecules involved in post-transcriptional gene regulation, with subsequent effects on various cellular and biological processes under normal and pathological conditions (11). Dysregulation of miRNA expression is a common feature observed in several human diseases, including cancer (12–14). In the context of tumorigenesis, miRNAs can act as tumor suppressors, through modulation of oncogenic cellular processes, or as oncogenes, through suppression of anti-tumor cellular pathways (15, 16). Altered miRNA expressions has been correlated with the pathogenesis of several human malignancies (17, 18), including breast carcinomas (19), making miRNAs as valuable diagnostic and prognostic molecular biomarkers (20–22). In the past decades, miRNAs have been intensively studied in primary breast tumor tissue leading to the identification of a number of diagnostic and prognostic miRNA signatures (23–25). Altered expression of several miRNAs, such as miR-7, miR-10a, miR-21, miR-126, miR-128, miR-205, miR-210, miR-484, miR-489, miR-494, and miR-548 have been reported in breast cancer (26–28). As minimally invasive disease biomarkers, our recent study have identified 18 diagnostic circulatory miRNA panel in breast cancer patients (22).

One limitation of published reports on LN-associated miRNAs in BC is that these studies are oftentimes conducted on different patient cohorts (29). In current study, we performed comprehensive miRNA profiling on 12 primary breast and their matched LN metastatic samples, which were subsequently validated in a second cohort of 32 breast and 32 matched LN tumor tissues. Functional and bioinformatics analyses highlighted novel insight into the role of miR-205-5p and miR-214-3p in suppressing BC LN metastasis.

## Materials and Methods

### Patient and Tissue Collection

Formalin-Fixed Paraffin-Embedded Tissue (FFPE) from 44 lymph node (LN) metastatic and matched primary breast samples was obtained from breast cancer patients undergoing standard treatment at the King Khalid University Hospital (Riyadh, Saudi Arabia); oncology center. Primary breast tumor and their paired Lymph node metastases were selected by an experienced pathologist from achieved FFPE sample blocks. Written informed consent was not required since the study was conducted on archived FFPE tissue blocks. Patient and tumor characteristics are listed in Table 1.

**Table 1:** Clinical information of patients included in current study and their tumor characteristics.

**Cohort I**	***N* = 44 primary and 44 LNM**	**%**
Age, y		
Median age	52 y	
Range	32–74 y	
Gender		
Female	44	100%
Stage		
I	0	00.0%
II	25	56.8%
III	14	31.8%
IV	5	11.4.0%
Molecular subtype		
Luminal	37	84.0%
HER2	3	06.8%
TN	4	09.0%
Hormone status		
ER	36	81.8%
PR	33	75.0%
HER2	10	22.7%

### Total RNA Isolation From Formalin-Fixed Paraffin-Embedded Tissue (FFPE)

Total RNA was extracted from FFPE sections using the recover all total nucleic acid isolation kit (Ambion Inc., Life Technologies, USA) according to the manufacturer's protocol with slight modifications. Briefly, 20-μm paraffin sections were cut, deparaffinized by incubation in xylene for 3 min at 50°C, centrifugation, and then washing twice in 100% ethanol. Subsequently, samples were washed and centrifuge at room temperature, and the pellets were vacuum-dried. Further, proteins were degraded using protease enzyme followed by 2–15 min incubations at 50 and 80°C and finally nucleic acid was purified using column before DNAse treatment. Finally, RNA was eluted in nuclease free water. The concentration and purity of total RNA was quantified using NanoDrop 2000 (Thermo Scientific, DE, USA).

### miRNA and Gene Expression Profiling by Microarray

Expression profiling of mRNA and miRNA was conducted as we previously described (30, 31). Initially, total RNA (including miRNA) was labeled and subsequently was hybridized to the Agilent SurePrint G3 Human GE 8 × 60 k or Human 8 × 60 k v21 miRNA microarray chip (Agilent Technologies, Palo Alto, CA, USA). Microarray experiments were performed at the Stem Cell Unit, Department of Anatomy, King Saud University College of Medicine. Raw data was then normalized and differential expression and pathway enrichment analyses were conducted using GeneSpring 13.0 software (Agilent Technologies). *P*-value < 0.05 and 2-fold cut-off were used. *In silico* target prediction was carried out using TargetScan release 7.2 database. Ingenuity Pathways Analysis (IPA; Ingenuity Systems; www.ingenuity.com/) was used for functional annotations and network analyses as we previously described (32).

### Taqman Low Density Array (TLDA) qRT-PCR

Custom TLDA miRNA cards were designed using unique microRNA primers and appropriate controls (RNU 44, RNU 48, and RNA U6) from Applied Biosystems. For miRNA cDNA synthesis, 500 ng of total RNA were used for reverse transcription employing Taqman microRNA reverse transcription kit (Applied Biosystems, CA, USA) according to the manufacturer's recommended protocol. CDNA was subsequently used for Taqman miRNA PCR amplification using custom TLDA cards and Taqman universal master mix no AmpErase UNG (Part No: 4324018). The samples were subjected to 40 cycles of 95°C for 10 min, 95°C for 15 s and 60°C for 60 s using ViiA7 Real-Time system (Applied Biosystems, CA, USA). The mean Ct-values were technically normalized using the endogenous control assay (RNU 44, RNU 48, and RNA U6), and the miRNA expression levels were presented as ΔCt.

### miRNA Transfection in MDA-MB-231-Cells

To investigate the functional role of selected miRNAs in regulating breast cancer biology, MDA-MB-231 cells (0.168 × 10^6^ cells/ml) were transfected with the selected miRNA precursors (pre-miR–negative control, hsa-miR-205-5p and hsa-miR-214-3p) purchased from Ambion. Cell transfection was conducted employing a reverse transfection protocol as we previously described (30, 33). Briefly, pre-miRs (at 30 nM final concentration) were diluted in 50 μl of Opti-MEM (Thermo Scientific, Rockford, IL, USA), and 1.5 μl of Lipofectamine 2000 (Thermo Scientific, Rockford, IL, USA) was diluted in 50 μl OPTI-MEM. The diluted pre-miRs, and Lipofectamine 2000 were then mixed and were incubated at room temperature for additional 20 min. Eight hundred microliters of transfection mixture were then transferred to the 6-well tissue culture plate and subsequently 0.168 × 10^6^ MDA-MB-231 cells/ml in transfection medium (OPTI-MEM) were added to each well. After overnight incubation, the transfection cocktail was replaced with fresh DMEM without antibiotics.

### Measurement of Cell Viability

The alamarBlue (Thermo Scientific, Rockford, IL, USA) assay was employed to assess cell viability as previously described (34). In brief, MDA-MB-231 cells (4 × 10^3^) transfected with pre-miR-205, pre-miR-214, and pre-miR-negative control cells were seeded in flat-bottom 96-well plates. Five days later, alamarBlue was added to each well at a final concentration of 10% and plates were subsequently incubated at 37°C for additional 2 h. The BioTek Synergy II (BioTek Inc., Winooski, VT, USA) plate reader was used to measure fluorescent intensity using 540 nM and 590 nM excitation and emission wavelengths, respectively. The cell viability of transfected breast cancer cell lines (miR-205 and miR-214) compared to pre-miR-negative control cells was presented as percent viability. Each experiment was done in a minimum of triplicates and was conducted at least twice.

### Colony Formation Assay

The colony forming ability of transfected MDA-MB-231 cells with miR-205-5p or hsa-miR-214-3p or pre-miR-negative control was assessed employing the clonogenic assay as we described before (31). In brief, transfected cells (4 × 10^4^) were seeded in 2 ml of DMEM in 12-well tissue culture plate. Subsequently, cells were serially diluted (1:1–1:32). The media was replaced with fresh media twice a week. Ten days later when colonies were formed, plates were washed and subsequently were stained with Diff-Quik Staining Kit (Siemens Healthcare Diagnostics, USA). The plates were then scanned and the number of formed colonies was counted.

### Transwell Migration Assay

MDA-MB-231 cells transfected with miR-205-5p, hsa-miR-214-3p, or pre-miR-negative control were serum-starved for 24 h in 1% serum media. For migration experiments, an 8.0 μm pore polyethylene terephthalate (PET, BD Falcon, MA, USA) inserts were employed as we described before (35). In brief, inserts were placed in 12-well notch plate, and subsequently cells transfected with the indicated miRNAs (5 × 10^4^) were resuspended in DMEM containing 1.0 % serum and were then added to the insert, while DMEM containing 10% serum was used as attractant in the lower chamber. Twelve and twenty four hours later, cotton swab was used to remove non-migrating cells, while migrated adherent cells on the lower surface of the membrane were stained using the Diff-Quik Stain. For quantitative analysis, the number of migrated cells from six random field was counted using microscope (Axio Observer-A1, Carl Zeiss, Germany).

### mRNA Validation by qRT-PCR

SYBR Green-based quantitative reverse transcriptase-polymerase chain reaction (qRT-PCR) was used to validate the relative expression of selected genes using the Applied Biosystems ViiA™ 7 Real-Time PCR System. Five hundred nanograms of total RNA was reverse transcribed to generate complementary DNA (cDNA) employing the High Capacity cDNA Reverse Transcript Kit (Thermo Scientific, Rockford, IL, USA). Relative mRNA expression was calculated using the 2^−ΔΔ*CT*^ method (36). β-actin housekeeping was used as endogenous control. Supplementary Table 1 lists the primer sequences used in current study.

### microRNA Analysis in Triple Negative Breast Cancer Cells

Raw miRNA sequencing data were obtained from sequence read archive (SRA) database under accession no. PRJNA423034 using the SRA toolkit version 2.9.2 as previously described (37). Small RNA sequencing was analyzed by first clipping the 3′ sRNA adapter and subsequent alignment and counting using CLC genomics workbench 12 (QIAGEN) and the miRBase 22 release database.

### Statistical Analysis

Statistical analyses were conducted using GraphPad Prism 8.0 software (GraphPad, San Diego, CA, USA). Two-tailed *t*-test was used to calculate significance. Unless stated otherwise, data are presented as mean ± standard error of the mean (S.E.M), from at least two independent experiments conducted in triplicate.

## Results

### Identification of Differentially Expressed microRNAs in Paired LN Metastatic and Primary Breast Cancer Tissue

**Figure 1:**
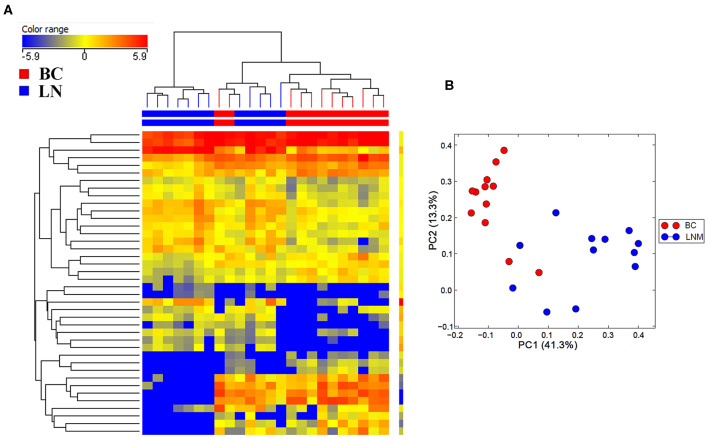
Hierarchical clustering depicting the expression of differentially expressed miRNAs in lymph node metastatic (LNM) and matched primary breast cancer (BC). **(A)** Hierarchical clustering of 12 LNM and 12 matched primary BC tissue based on differentially expressed miRNA levels. Each column represents a sample and each row represents a miRNA. Expression level of each miRNA in a single sample is depicted according to the color scale. **(B)** Principal component analysis (PCA) for the miRNA transcriptome of 12 LNM and their matched BC tissue.

**Figure 2:**
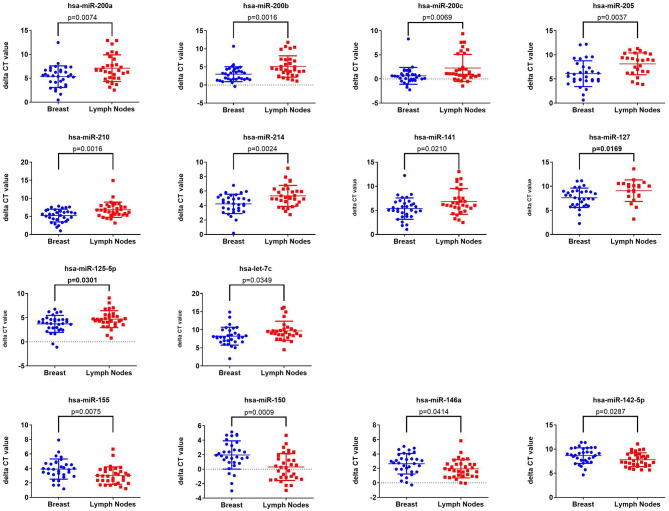
Validation of the expression of 14 miRNAs identified from microarray data in a second cohort of 32 LNM and 32 matched primary BC tissue Taqman low density array (TLDA) qRT-PCR. *P*-values were calculated using two-tailed *t*-test and are indicated on each plot. Data are presented as “delta CT” using dot plots.

Initial profiling was conducted on 24 samples of BC primary and matched LN metastatic derived from 12 BC patients. Patients and tumor characteristics are summarized in Table 1. A total of 660 miRNAs were detected in BC and LN tissue (Supplementary Table 2). Using Benjamini-Hochberg False Discovery Rate (FDR) multiple testing correction [*p*_(corr)_ < 0.05] method and 2-fold change cut-off, we identified 40 miRNAs differentially expressed between LN metastatic and primary breast cancer tissue (Table 2). Hierarchical clustering based on differentially expressed miRNAs revealed clear separation of the two groups, except for two primary BC samples which clustered with the LN group (Figure 1). The expression levels of 10 downregulated (hsa-miR-200a-3p, hsa-miR-200b-3p, hsa-miR-200c-3p, hsa-miR-205-5p, hsa-miR-210-3p, hsa-miR-214-3p, hsa-miR-141-3p, hsa-miR-127-3p, hsa-miR-125a-5p, and hsa-let-7c-5p) and four upregulated (hsa-miR-155-5p, hsa-miR-150-5p, hsa-miR-146a-5p, and hsa-miR-142-5p) were subsequently validated in a second cohort of 32 LN metastatic and their matched 32 primary breast cancer tissues using custom TLDA miRNA cards, which was concordant with the microarray data (Figure 2).

**Table 2:** Differentially expressed miRNAs comparing LN and matched primary breast cancer tumors.

**Systematic name**	**mirbase accession no**	***p*_**(Corr)**_**	**Regulation**	**FC**
hsa-miR-142-5p	MIMAT0000433	0.026	up	60.317852
hsa-miR-6834-3p	MIMAT0027569	0.031	up	18.272284
hsa-miR-4455	MIMAT0018977	0.042	up	16.318874
hsa-miR-3151-3p	MIMAT0027026	0.028	up	13.968794
hsa-miR-363-3p	MIMAT0000707	0.039	up	13.691544
hsa-miR-150-5p	MIMAT0000451	0.006	up	13.24406
hsa-miR-6716-3p	MIMAT0025845	0.039	up	9.201071
hsa-miR-4436b-5p	MIMAT0019940	0.043	up	8.730566
hsa-miR-146a-5p	MIMAT0000449	0.015	up	7.50569
hsa-miR-328-3p	MIMAT0000752	0.043	up	7.0950775
hsa-miR-155-5p	MIMAT0000646	0.006	up	4.67067
hsa-miR-505-3p	MIMAT0002876	0.045	up	4.235982
hsa-miR-8485	MIMAT0033692	0.039	up	3.0183256
hsa-miR-140-3p	MIMAT0004597	0.003	up	2.7240493
hsa-miR-146b-5p	MIMAT0002809	0.037	up	2.6890745
hsa-miR-222-3p	MIMAT0000279	0.006	up	2.5138452
hsa-miR-223-3p	MIMAT0000280	0.031	up	2.4507039
hsa-miR-664b-3p	MIMAT0022272	0.006	up	2.4268947
hsa-miR-361-3p	MIMAT0004682	0.013	up	2.136327
hsa-miR-3653-3p	MIMAT0018073	0.006	up	2.0807796
hsa-let-7a-5p	MIMAT0000062	0.028	down	−2.081669
hsa-let-7b-5p	MIMAT0000063	0.013	down	−2.1661768
hsa-let-7e-5p	MIMAT0000066	0.011	down	−2.1978226
hsa-miR-10a-5p	MIMAT0000253	0.034	down	−2.204046
hsa-miR-125a-5p	MIMAT0000443	0.010	down	−2.2378376
hsa-miR-99a-5p	MIMAT0000097	0.010	down	−2.4249141
hsa-let-7c-5p	MIMAT0000064	0.003	down	−2.5129604
hsa-miR-199b-5p	MIMAT0000263	0.015	down	−3.0455856
hsa-miR-214-3p	MIMAT0000271	0.048	down	−3.7974756
hsa-miR-210-3p	MIMAT0000267	0.044	down	−6.5551286
hsa-miR-370-3p	MIMAT0000722	0.041	down	−7.664438
hsa-miR-376a-3p	MIMAT0000729	0.046	down	−12.530114
hsa-miR-375	MIMAT0000728	0.037	down	−22.364883
hsa-miR-127-3p	MIMAT0000446	0.006	down	−22.525473
hsa-miR-429	MIMAT0001536	0.014	down	−31.980312
hsa-miR-200b-3p	MIMAT0000318	0.028	down	−50.233257
hsa-miR-200a-3p	MIMAT0000682	0.011	down	−53.79745
hsa-miR-141-3p	MIMAT0000432	0.034	down	−57.286835
hsa-miR-200c-3p	MIMAT0000617	0.026	down	−100.81091
hsa-miR-205-5p	MIMAT0000266	0.028	down	−117.2405

### Forced Expression of hsa-miR-205-5p and hsa-miR-214-3p Reduces MDA-MB-231 Cell Growth, Migration and Colony Formation

**Figure 3:**
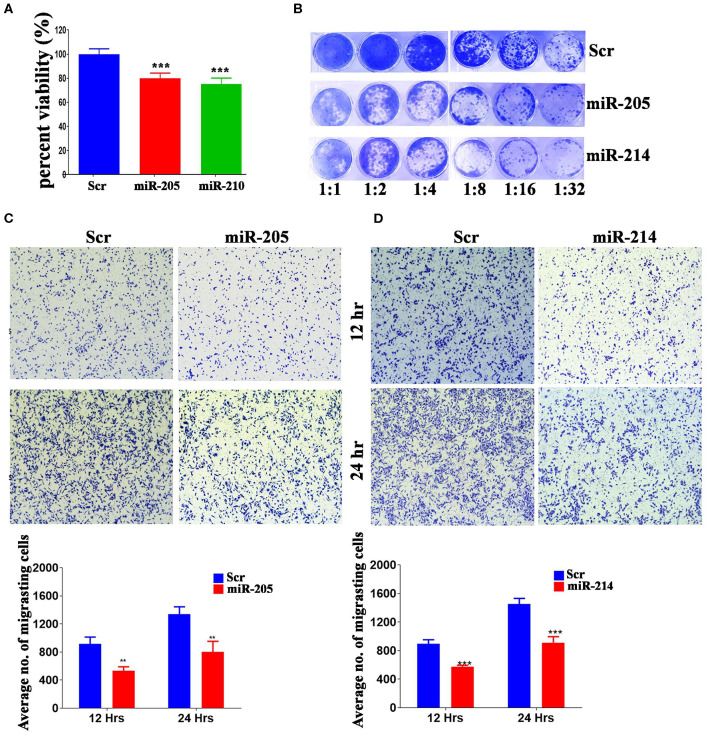
MiR-205-5p and miR-214-3p inhibits MDA-MB-231 BC cell proliferation, colony formation and migration. **(A)** Cell viability of MDA-MB-231 cells transfected with hsa-miR-205, hsa-miR-214-3p, or scrambled-control measured on day 5 using alamarblue assay. Data are presented as mean ± S.E.M., *n* = 6. **(B)** Clonogenic assay showing the colony forming capability of MDA-MB-231 cells transfected with hsa-miR-205, hsa-miR-214-3p, or scrambled-control. Plates were stained with Diff-Quik stain set on day 10. Wells are representative of two independent experiments for each condition. **(C,D)** Migration capability of MDA-MB-231 cells transfected with hsa-miR-205, hsa-miR-214-3p compared to scrambled-control. The two-tailed *t*-test was used to compare different treatment groups. **p < 0.01; ***p < 0.001.

Several of the identified miRNAs were previous reported to regulate BC cell biology (38). Therefore, we subsequently investigated the biological role for the less studied, hsa-miR-205-5p and hsa-miR-214-3p, miRNAs in regulating BC function. These experiments were conducted on the highly metastatic MDA-MB-231 model, which expresses those miRNAs at low levels (Supplementary Figure 1). As presented in Figure 3, forced expression of hsa-miR-205-5p and hsa-miR-214-3p exhibited remarkable inhibition of cell proliferation, colony formation and cell migration of MDA-MB-231 breast cancer cells (Figures 3A–D), hence corroborating a role for those miRNAs in driving BC metastasis.

### Identification of hsa-miR-205-5p and hsa-miR-214-3p Bone Fide Gene Targets in BC Cells

**Figure 4:**
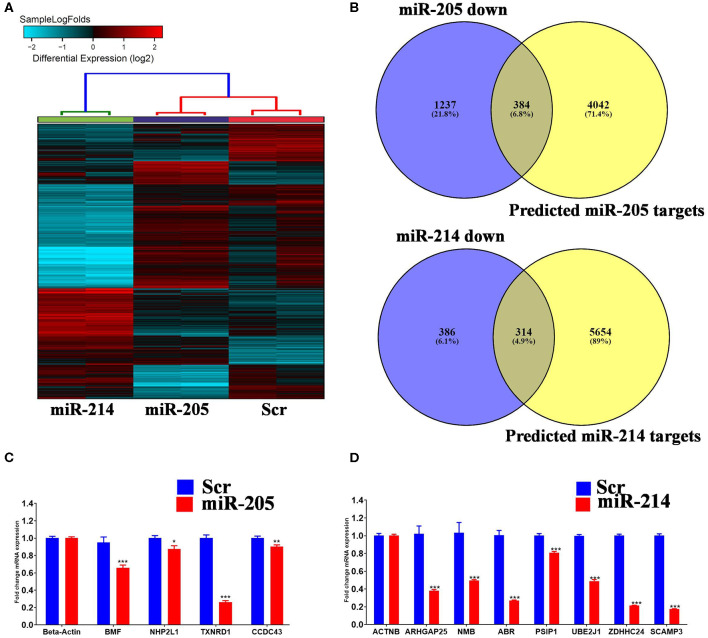
Identification of miR-205-5p and miR-214-3p *bona fide* gene targets in BC cells. **(A)** Hierarchical clustering on differentially expressed transcripts in MDA-MB-231 cells transfected with hsa-miR-205, hsa-miR-214 compared to scramble control. Each column represents one replica and each row represents a transcript. Expression level of each gene in a single sample is depicted according to the color scale. **(C,D)** Venn diagram depicting the overlap between the predicted gene targets for hsa-miR-205-5p or hsa-miR-214-3p (based on TargetScan algorithm) and the downregulated genes in MDA-MB-231 cells overexpressing the respective miRNA. **(B)** The expression levels of selected genes targets for hsa-miR-205-5p or hsa-miR-214-3p were validated using qRT-PCR in MDA-MB-231 cells overexpressing the respective miRNA. Data are presented as mean ± S.E., *n* = 6. The two-tailed *t*-test was used to compare different treatment groups. *p < 0.05; **p < 0.01; ***p < 0.001.

In order to gain more insight into the mechanism by which those two miRNAs regulate BC cell function, global gene expression profiling was conducted on MDA-MB-231 cells over expressing hsa-miR-205-5p and hsa-miR-214-3p compared to negative control cells. Hierarchical clustering revealed different pattern of gene expression in MDA-MB-231 cells under various treatment conditions (Figure 4A). Among the experimentally downregulated genes in MDA-MB-231 cells transfected with hsa-miR-205-5p, 384 genes were predicted as hsa-miR-205-5p targets based on in silicon prediction (Figure 4B, upper panel). Similarly, among the experimentally downregulated genes in MDA-MB-231 cells transfected with hsa-miR-214-3p, 314 genes were predicted as hsa-miR-214-3p targets based on in silicon prediction (Figure 4B, lower panel). The expression of selected number of hsa-miR-205-5p and hsa-miR-214-3p gene targets was validated using qRT-PCR (Figures 4C,D), corroborating the microarray data.

### Multiple Regulated Cellular Pathways by hsa-miR-205-5p and hsa-miR-214-3p in BC Cells

**Figure 5:**
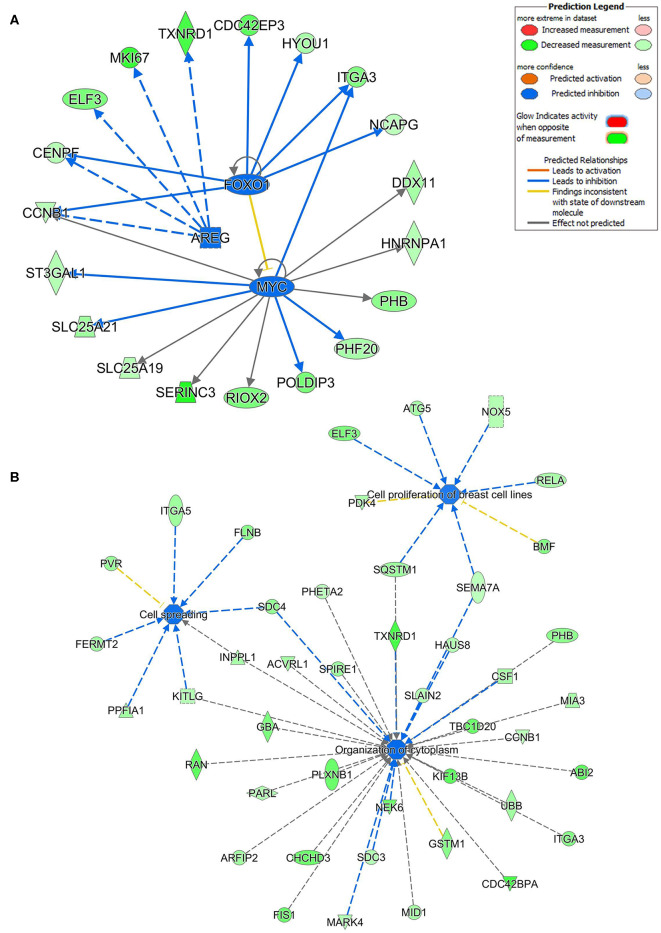
Multiple signaling networks are regulated by hsa-miR-205-5p based on ingenuity pathway analysis (IPA). **(A)** Illustration of the top inhibited mechanistic networks (FOXO1, AREG and MYC) based on the identified hsa-miR-205-5p gene targets and IPA analysis. **(B)** Functional networks illustrating the involvement of the indicated hsa-miR-205-5p gene targets in regulating cell proliferation, cell spreading, and organization of cytoskeleton functional based on IPA analysis.

**Figure 6:**
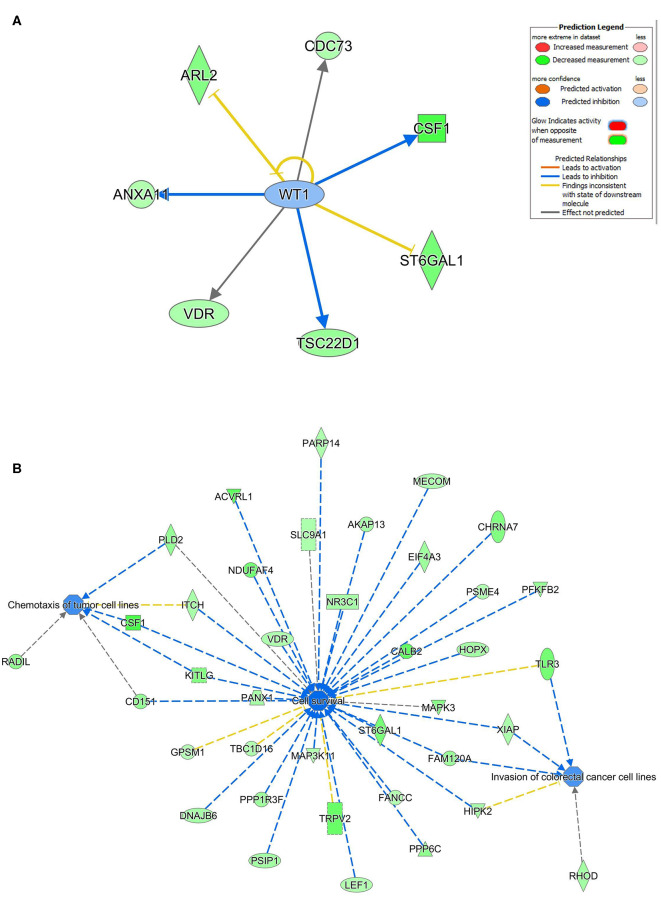
Regulated of the WT1 network, cell survival, invasion, and chemotaxis by hsa-miR-214. **(A)** Illustration of the WT1 mechanistic network based on the identified hsa-miR-214-3p gene targets and IPA analysis. **(B)** Functional networks illustrating the involvement of the indicated hsa-miR-214-3p gene targets in regulating cell proliferation, cell spreading, and organization of cytoskeleton based on IPA analysis.

Ingenuity pathway analysis on validated hsa-miR-205-5p gene targets revealed suppression of several mechanistic networks by hsa-miR-205-5p in BC cells. In particular, the MYC, FOXO1, and AREG networks were most affected by hsa-miR-205-5p (Figure 5A). Notably, 12 gene targets (ITGA3, DDX11, NRNPA1, PHB, PHF20, POLDIP3, RIOX2, SERINC3, SLC25A19, SLC25A21, ST3GAL1, and CCNB1) were associated with the MYC, six genes (NCAPG, ITGA3, HYOU1, CDC42EP3, CENPF, and CCNB1) were associated with the FOXO1, and five genes (TXNRD1, MKI167, ELF3, CENPF, and CCNB1) were associated with the AREG signaling networks. Several of the identified hsa-miR-205-5p gene targets were also involved in regulating cell proliferation, cell spreading, and organization of cytoskeleton functional categories, all associated with BC metastatic phenotype (Figure 5B). On the other hand, IPA revealed hsa-miR-214-3p to mainly regulate the WT1 networks (Figure 6A). Seven gene targets (VDR, TSC22D1, ST6GAL1, CSF1, CDC73, ARL2, and ANXA11) were connected to the WT1 network. Concordantly, several of the identified hsa-miR-214-3p targets were found to regulate cell survival, invasion, and chemotaxis, corroborating a role for this miRNA in driving BC metastasis (Figure 6B).

### miR-205 and miR-214 Expression Correlates With Worse Prognosis of BC Patients

**Figure 7:**
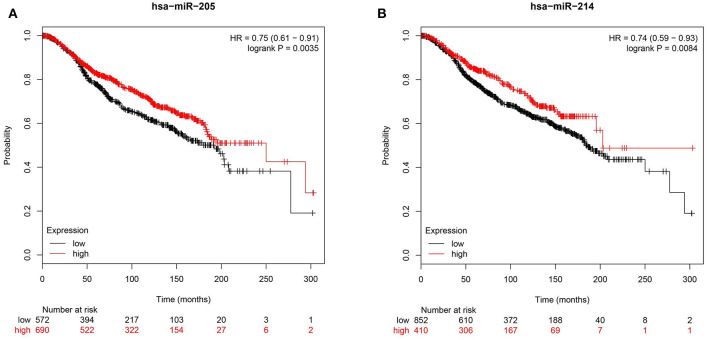
Reduced expression of hsa-miR-205-3p or hsa-214-3p is associated with poor prognosis in BC patients. Kaplan-Meier curves illustrating the duration of overall survival (OS) of 1,262 BC patients stratified into high vs. low based on hsa-miR-205 (HR = 0.75, *p* = 0.003, **A**) or hsa-miR-214 (HR = 0.74, *p* = 0. 008, **B**) expression from the METABRIC BC dataset.

To further corroborate clinical role for miR-205 and miR-214 in BC, we investigated the correlation between miR-205 and miR-214 expression and BC patients' survival in the METABRIC (39) cohort consisting of 1,262 BC patients. Survival data revealed elevated miR-205 and miR-214 expression as favorable prognostic markers in breast cancer [HR = 0.75 (0.61–0.91)], *p* = 0.003 for miR-205 and HR = 0.74 (0.59–0.93) *p* = 0.008 for miR-214 (Figure 7).

## Discussion

Although regional lymph node metastasis found at the time of primary diagnosis is curable using surgery and radiotherapy, such regional metastasis confers poor prognostic factor, and is oftentimes associated with a higher risk of subsequent cancer recurrence. While the bulk of genomic and transcriptome analysis have been devoted to primary breast cancers, data available for metastatic breast cancer cells revealed the existence of similar genomic alterations in metastatic cells similar to those seen in primary tumors, in addition to the presence of *de novo* acquired alterations (40). Currently there are limited resources on the microRNA expression profile of primary and matched metastasis BC tissue especially from this region. In current study, we are the first to conduct global miRNA expression profiling on primary breast cancer and matched lymph node metastatic tissue from this geographic region. Through transcriptome analysis, functional and pathway analyses, our data implicated hsa-miR-205-5p and hsa-miR-214-3p in breast cancer LN metastasis, through regulation of a number of oncogenic networks.

Among the 660 expressed miRNAs, only 40 miRNAs were differentially expressed in primary compared to LN metastatic BC cells, suggesting the acquisition of metastatic phenotype could be driven by a handful of miRNAs. The expression of several of the identified miRNAs was subsequently validated in a second cohort of primary BC and matched LN metastasis. Interestingly, several of the downregulated miRNAs in LN metastasis were previously linked to BC stem cells. In particular, downregulated expression of several members of the miR-200 family (hsa-miR-200a, hsa-miR-200b, and hsa-miR-200c) was validated in LN metastatic tumors. Interestingly, a number of previous studies have highlighted a role for MiR-200 family in BC biology. miR-200 members were found to regulate BMI1 expression in breast cancer tumor initiating cells and also to suppress epithelial mesenchymal transition (EMT) through inhibition of zinc-finger E-box binding homeobox (ZEB)1 and ZEB2 and subsequent modulation of BC cell migration (41–43). Additionally, miR-200c was shown to regulate transforming growth factor β-induced stress fiber formation through targeting formin homology 2 domain containing (FHOD)1, an actin-regulatory proteins, and protein phosphatase, Mg^2+^/Mn^2+^-dependent (PPM)1F (43). Taken together, it is plausible that BC LN metastasis is driven in part through loss of miR-200 family and subsequent upregulation of BMI1 and enrichment in BC TI-Cs, upregulation of ZEB1, ZEB2, and PPM1F and subsequent acquisition of an EMT phenotype.

To provide mechanistic insight into miRNA and LN metastasis, the highly metastatic MDA-MB-231 model was used for miR-205-5p and miR-214-3p functional and mechanistic investigations. Forced expression of miR-205-5p and miR-214-3p suppressed BC cell proliferation, migration, and colony formation suggesting a plausible role for those miRNAs in regulating BC metastatic. Network and functional annotation analysis on the identified miR-205-5p and miR-214-3p gene targets revealed potential regulation of several oncogenic networks in BC cells, hence suppressing cytoskeleton organization, cell proliferation, and migration, and chemotaxis. In particular, miR-205-5p was found to regulate MYC, FOXO1, and AREG networks in BC. Concordant with mechanistic data, lower expression of hsa-miR-205-5p and hsa-miR-214-3p were associated with worse overall survival in 1,262 BC patients from the METABRIC cohort. Our data are in agreement with other reports implicating miR-205 in regulating EMT (44) and as favorable prognostic marker in colorectal (44) and head and neck cancers (45). In summary, our data have identified several miRNAs associated with LN metastasis in BC patients and provided mechanistic insight into regulation of cell proliferation, and migration by hsa-miR-205-5p and hsa-miR-214-3p and their potential involvement in BC LN metastasis for potential utilization as prognostic biomarkers and targets for therapeutic interventions.

## Data Availability Statement

The original contributions presented in the study are publicly available. This data can be found here: the NCBI Gene Expression Omnibus (GSE148847, GSE148848).

## Ethics Statement

The studies involving human participants were reviewed and approved by the Institutional Research Ethics Board at the King Saud University College of Medicine. Written informed consent for participation was not required for this study in accordance with the national legislation and the institutional requirements.

## Author Contributions

RE performed experiment and participated in manuscript writing. RV and MM performed experiments. KA involved in conception and design, in patient selection and clinical interpretation, and obtained funding. AMA, NAE-A, and AAlt involved in patient selection and clinical data interpretation. AA-R involved in pathological examination. MA and AAld involved in conception and design. NMA obtained funding, conceive the study, analyzed data, and finalized manuscript.

## Conflict of Interest

The authors declare that the research was conducted in the absence of any commercial or financial relationships that could be construed as a potential conflict of interest.
