# Supplementary material for: MicroRNA Expression Profiling on Paired Primary and Lymph Node Metastatic Breast Cancer Revealed Distinct microRNA Profile Associated With LNM
Source: Front Oncol. 2020 May 19;10:756. doi: 10.3389/fonc.2020.00756 (PMC7248321; doi:10.3389/fonc.2020.00756)
Supplement: Supplementary file 1 [file Table_1.doc]

| **Supplementary table 1. Primer sequences used in the current study** | | | | |
| --- | --- | --- | --- | --- |
| **S.No** | **Name of Primers** | **Forward Sequences** | **Reverse Sequences** | **Accession No** |
| 1 | β–Actin | TGTGCCCATCTACGAGGGGTATGC | GGTACATGGTGGTGCCGCCAGACA | NM_001101.5 |
| 2 | BMF | TTCCCTCCTTCCCAATCGAG | CATCTCTCCTGGGTGACTCC | NM_001003940.1 |
| 3 | NHP2L1 | GCGAGTGCTTCTGAAACGTC | TTGGTCCCAGGACAACATGG | NM_005008.3 |
| 4 | TXNRD1 | CAGTTTGCAGAACAGCGGAG | GCCCTCCTGATAAGCCTTCAA | NM_001261445.1 |
| 5 | C16ORF52 | TTTCTGGCCGCCGATATCTT | GTGAGTGCTCCCGCAGAC | NM_001164579.1 |
| 6 | CCDC43 | TGGCCCAGTATGCTGATGTG | GGAACAGAAGTTTGTCAGAACCA | NM_144609.2 |
| 7 | ARHGAP25 | AACCCAGAAGAAGCTGGGAA | CAAACACTGCTCCACAGGG | NM_001007231.2 |
| 8 | NMB | TGGGCAAGAAGAGTCTGGAG | TCCTGTACTGGATTTGGGGTG | NM_021077.3 |
| 9 | ABR | TTTCTCCTGGAGGAGTGCG | TTTCCCTGCTTCCACCTTCA | NM_001092.4 |
| 10 | PSIP1 | ACGCAAGCAAGAGGAACAAA | TCAATGCATCTGTTCACATCAAGA | NM_001128217.2 |
| 11 | UBE2J1 | GCCAGTACATCGTACGGACTC | TCGAGGGCTCATGGAGGTAT | NM_016021.2 |
| 12 | ZDHHC24 | CCTGCTCCACGTCTCTGTG | GACACTCTGCCTGTGAGCAA | NM_207340.2 |
| 13 | SCAMP3 | GACAACCCCTTTCAGGACCC | AGGGTTGTAGACGTCAAGCG | NM_005698.3 |
