# Supplementary material for: MicroRNA Expression Profiling on Paired Primary and Lymph Node Metastatic Breast Cancer Revealed Distinct microRNA Profile Associated With LNM
Source: Front Oncol. 2020 May 19;10:756. doi: 10.3389/fonc.2020.00756 (PMC7248321; doi:10.3389/fonc.2020.00756)
Supplement: Supplementary file 3 [file Data_Sheet_1.docx]

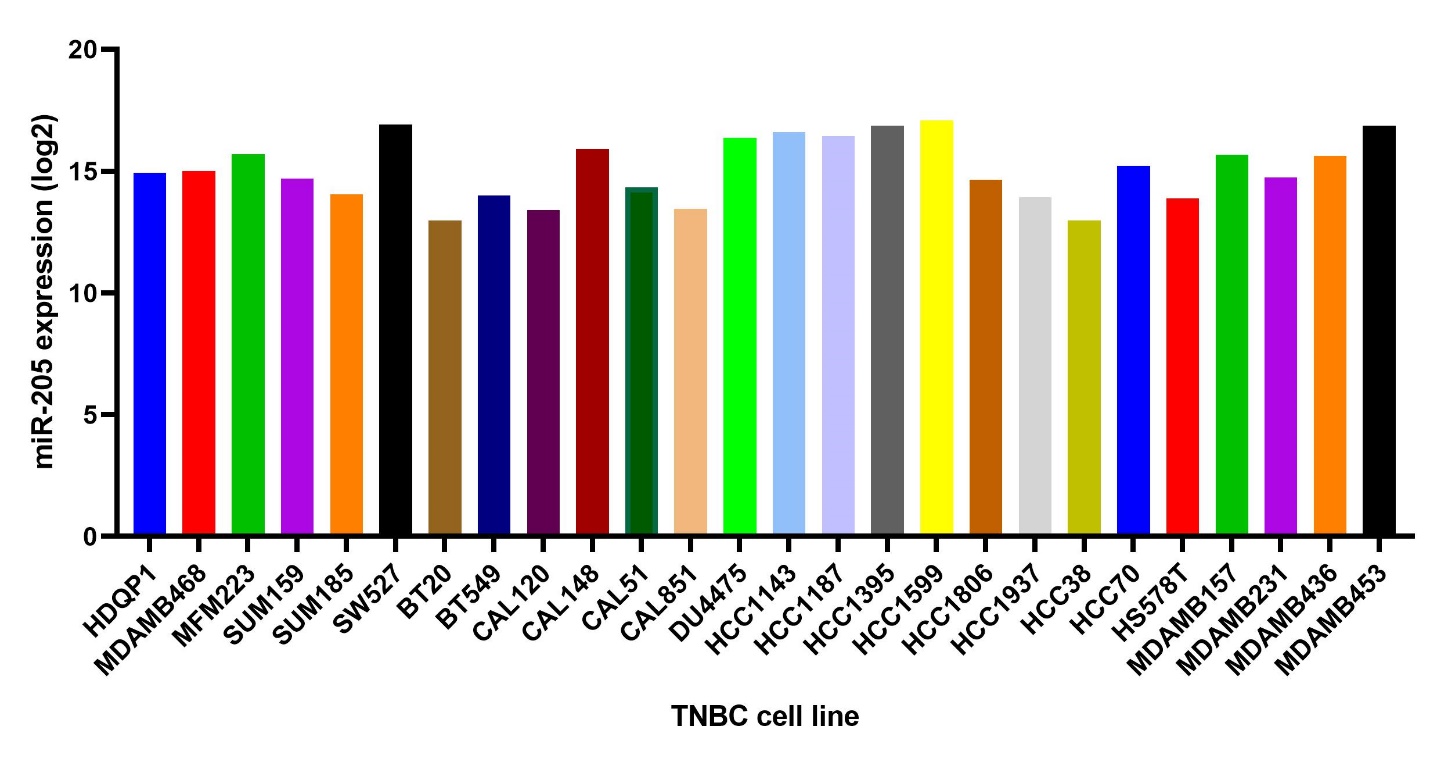


**Supplementary figure 1.** MiR-205 expression in a panel of TNBC cell lines based on RNASEQ data analysis from the PRJNA423034 project.
